# Comparison of grain traits and genetic diversity between Chinese and Uruguayan soybeans (*Glycine max* L.)

**DOI:** 10.3389/fpls.2024.1435881

**Published:** 2024-07-24

**Authors:** Chang Sun, Zhihao Zhang, Meiling Liu, Sergio Ceretta, Shengrui Zhang, Bingfu Guo, Yinghui Li, Zhangxiong Liu, Yongzhe Gu, Xue Ao, Lijuan Qiu

**Affiliations:** ^1^ College of Agronomy, Shenyang Agricultural University, Shenyang, China; ^2^ The National Key Facility for Crop Gene Resources and Genetic Improvement (NFCRI)/State Key Laboratory of Crop Gene Resources and Breeding/Key Laboratory of Crop Gene Resource and Germplasm Enhancement (MOA)/Key Laboratory of Grain Crop Genetic Resources Evaluation and Utilization, Institute of Crop Science, Chinese Academy of Agricultural Sciences, Beijing, China; ^3^ Key Laboratory of Soybean Biology in Chinese Ministry of Education (Key Laboratory of Soybean Biology and Breeding/Genetics of Chinese Agriculture Ministry), Northeast Agricultural University, Harbin, China; ^4^ National Agricultural Research Institute (INIA), Soybean Breeding Program, Colonia, Uruguay; ^5^ The National Engineering Research Center for Crop Molecular Breeding, Ministry of Agriculture and Rural Affairs (MARA) Key Laboratory of Soybean Biology (Beijing), Institute of Crop Sciences, Chinese Academy of Agricultural Sciences, Beijing, China; ^6^ Nanchang Branch of the National Center of Oilcrops Improvement, Jiangxi Province Key Laboratory for the Genetic Improvement of Oilcrops, Institute of Crops, Jiangxi Academy of Agricultural Sciences, Nanchang, China

**Keywords:** soybean, Uruguay, oil content, genetic structure, genetic diversity

## Abstract

Soybeans (*Glycine max* L.), originating in China, were introduced to South America in the late 19th century after passing through North America. South America is now a major soybean-producing region, accounting for approximately 40% of the global soybean production. Crops like soybeans gradually adapt to the local climate and human-selected conditions, resulting in beneficial variations during cultivation in different regions. Comparing the phenotypic and genetic variations in soybeans across different regions is crucial to determining the variations that may enhance soybean productivity. This study identified seed-related traits and conducted a genetic diversity analysis using 46 breeding soybean varieties from China and Uruguay. Compared to the Chinese soybean germplasm, the Uruguayan equivalent had a lower 100-grain weight, higher oil content, lower protein content, and higher soluble sugar content. Using ZDX1 gene chips, genetic typing was performed on the 46 breeding varieties. Cluster analysis based on SNP sites revealed significant differences in the genetic basis of Sino-Uruguayan soybean germplasm. Selection analysis, including nucleotide polymorphism (π) and fixation indexes (Fst), identified several genomic regions under selection between Sino-Uruguayan soybean germplasm. The selected intervals significantly enriched gene ontology (GO) terms related to protein metabolism. Additionally, differentiation occurred in genes associated with the oil content, seed weight, and cyst nematodes between Sino-Uruguayan soybean germplasm, such as *GmbZIP123* and *GmSSS1*. These findings highlight the differences in seed-related phenotypes between Sino-Uruguay soybean germplasm and provide genomic-level insights into the mechanisms behind phenotypic differences, offering valuable references for understanding soybean evolution and molecular breeding.

## 1 Introduction

Soybeans (*Glycine max* L.), originating from China (Dong et al., 2022), have a long history of cultivation dating back 5,000 years (Qu et al., 2023). Soybeans are rich in plant proteins and oils. Seed proteins and oils are crucial factors that determine the nutritional and economic value of soybeans (Patil et al., 2017). In the mid to late 18th century, soybeans were introduced to North America (Hymowitz and Shurtleff, 2005). By the end of the 19th century, soybeans had been transferred from North to South America (Pregnolatto, 1981). Despite the relatively brief history of soybean cultivation in South America, this region is currently one of the world’s most significant soybean-producing areas. In terms of soybean variety improvements, South America has managed to develop new soybean varieties with high yields stability and adaptability (Wang, 2014). In Uruguay, soybeans occupy more than half of the total cultivated cereal area and greatly contribute to export revenue (DIEA, 2020; OPYPA, 2020).

Germplasm resources are the foundation for breeding new varieties and conducting fundamental research. The richer the soybean germplasm resources, the greater the potential to develop superior varieties (Zhou et al., 2022). Soybean germplasm is jointly regulated by genetic and environmental factors, resulting in a diverse range of phenotypic variations. One of the primary contributing factors to soybean yield is the 100-grain weight (Lu et al., 2017). Controlled by multiple genes and possessing a high heritability, the 100-grain weight is significantly influenced by environmental factors (Mian et al., 1996). Soybean quality characteristics are typically associated with traits such as seed oil, protein, and sugar contents (Teng et al., 2017; Zhang Q. et al., 2023). The protein and oil content of soybeans are typical phenotypes resulting from the interaction between genetic factors and the environment (Pathan et al., 2013). Research indicates that during the cultivation of soybeans in different regions, changes in seed traits have occurred due to varying environmental factors and breeding objectives. A comparison of 277 Chinese soybean varieties and 300 American soybean varieties found that American soybean varieties are characterized by taller plants, more branches, more pod numbers, and higher yields (Liu Z. et al., 2017). Studies have revealed a significant negative correlation between soybean protein and oil contents (Bandillo et al., 2015), which poses a challenge when attempting to improve the soybean oil content and yield while maintaining the desired protein content. The total and soluble sugar contents of soybean germplasm also exhibit rich genetic variations, with substantial differences in the soluble sugar content observed in different environments (Lu et al., 2022). This variability can provide valuable germplasm resources for breeding (Geater and Fehr, 2000).

*GmMFT* participates in the regulation of the seed oil content, with different alleles of this gene significantly impacting the oil content (Cai et al., 2023). Analysis of 46 Sino-Uruguayan soybean germplasm revealed differentiation in the frequency of this gene between the AA and CC regions. Additionally, *GmDGAT1b* increases the oleic acid levels, thereby affecting the fatty acid composition of the seeds (Torabi et al., 2021). Divergence in the allele frequencies of *GmDGAT1b* in the AA and CC regions gives rise to significant differences in the oil contents of Sino-Uruguayan soybean germplasm. Overexpression of *GmWRKY12* enhances the resistance of transgenic soybeans to salt and drought (Shi et al., 2018). Similarly, differentiation in the allelic gene frequency of *GmWRKY12* between the AA and GG regions in Sino-Uruguayan soybean germplasm is the primary cause for the observed differences in drought-related traits between these germplasms. *GmFT2a* has been shown to facilitate flowering through transgenic studies in both Arabidopsis and soybeans (Sun et al., 2011). Analysis of 46 Sino-Uruguayan soybean germplasm revealed differentiation in the allelic frequencies of *GmFT2a* in the GG and TT regions, leading to variations in the flowering phenotype.

Utilization of the complementary advantages of germplasm from different regions will provide abundant breeding materials to develop high-yield, high-quality, and environmentally adaptive varieties. This is of paramount importance to enhancing soybean production in China and ensuring global food security. Therefore, this study leveraged valuable germplasm resources from Uruguay, a key soybean producer in South America. Through a comparison of the seed characteristics and genetic diversity of 46 Sino-Uruguayan soybean germplasm, genes related to seed quality characteristics, including *GmbZIP123*, *GmMFT*, *GmDGAT1b*, and *GmSSS1*, were identified. This research offers valuable information of genetic resources to improve soybean yield and quality.

## 2 Materials and methods

### 2.1 Plant materials and experimental design

A total of 46 soybean germplasm (**Supplementary Table 1**), including 22 from China and 24 from Uruguay, were provided by the Crop Science Institute of the Chinese Academy of Agricultural Sciences. In 2022, all germplasms were planted in the Tacheng Experimental Station (Nanchang, Jiangxi Province); and seeds were sown on July 22^nd^. Triplicates were sown for each seed, and replicates were planted across three rows, with each being 2 meters in length. Row spacing was 40 cm, and plant spacing was 8 cm. The 1,856 soybean germplasm for this comparative analysis were planted in Hainan on November 16^th^, 2018. The planting site was located at the Sanya Experimental Station of the Crop Science Institute, in the Chinese Academy of Agricultural Sciences. Cultivation conditions were 35 cm row spacing, 10 cm plant spacing, and normal water and fertilizer management. Material genotype data were obtained from Li et al. (2023).

### 2.2 Phenotypic identification

A total of 100 seeds were randomly selected for seed weight measurement and the triplicate averages were calculated. Approximately 50 g of undamaged seeds were randomly selected, and the protein and oil contents were measured using Fourier-transform near-infrared spectroscopy (FT-NIR). Soluble sugar measurements were performed using the method described by Agyenim-Boateng et al. (2023). A total of 100 mg of soybean powder was mixed with 50% acetonitrile, and the samples were shaken for 8h at room temperature in an incubator. Then 500 mL of the supernatant was transferred to a new tube containing 200 μL of acetonitrile, and the mixture was shaken to achieve protein precipitation. After 10 min at room temperature, the samples were centrifuged at 20°C for 10 min, and the supernatant was filtered using a syringe filter (0.22 μm) before detection using a UPLC-RID (Agyenim-Boateng et al., 2023).

### 2.3 DNA extraction and SNP genotyping

DNA extraction and SNP analysis were conducted by the Beijing Comsen Biological Technology Co., Ltd., in collaboration with the Crop Science Institute of the Chinese Academy of Agricultural Sciences, using the co-developed “Zhongdouxin-1” chip (Sun et al., 2022). After genotyping, Tianjin Jizhi Genes Technology Co., Ltd. obtained the SNP genotype data for all samples. SNPs were filtered based on a minimum allele frequency (MAF) < 0.05 and missing rate (Miss) > 0.8, resulting in a total of 96,446 SNPs that were used for subsequent analysis. Genomic variation of the natural population was analyzed in a previous study (Li et al., 2023).

### 2.4 Candidate gene analysis

The soybase database (https://www.soybase.org/) as a reference genomic map was searched to identify candidate genes. Based on the linkage disequilibrium (LD) decay distance in soybeans, a gene scan was performed within 100 kb upstream and downstream of significant sites, using the physical positions of all SNP sites, to identify genes affecting the phenotypic traits of the Sino-Uruguayan soybean varieties.

### 2.5 Statistical data analysis

Of the 46 soybean samples, 22 were from China and 24 were from Uruguay, with a total of 96,446 SNPs for subsequent analysis. The distance matrix was computed using TreeBeST (version: Treebest-1.9.2) software in order to construct a phylogenetic tree, with bootstrap values obtained through 1000 iterations. Principal component analysis (PCA) was conducted by Jizhi Genes using GCTA (Yang et al., 2011), and Admixture (version 1.3.0) (Alexander et al., 2009) was used for population structure analysis, analyzed separately for k=2 and k=3. The genetic diversity parameter (Pi) and fixation index (Fst) were calculated using VCFtools (Danecek et al., 2011), with a window size of 10 kb and a step size of 5 kb. Pop LDdecay software (Zhang C. et al., 2019) was used to analyze the LD, half of the LD decline to the maximum value is the LD decay distance.

## 3 Results

### 3.1 Phenotypic trait differences

**Figure 1:**
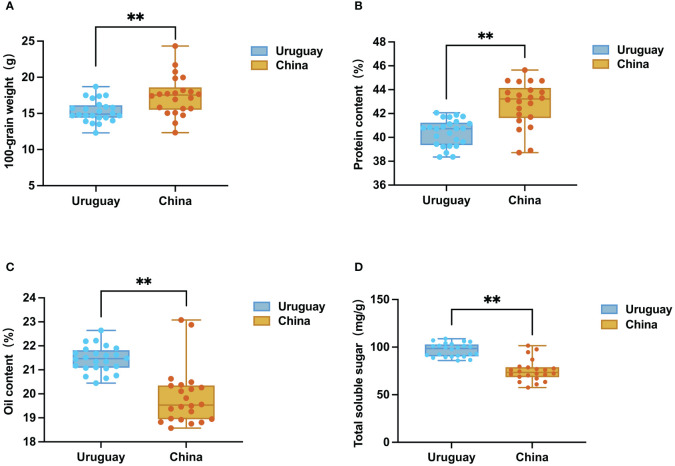
Box plots of grain traits of cultivars from Uruguay and China. **(A)** Distribution of 100-grain weight. **(B)** Distribution of protein content. **(C)** Distribution of oil content. **(D)** Distribution of total soluble sugar. **, P <0.01, according to Student’s t-test.

Analysis of the 46 Sino-Uruguayan soybean germplasm revealed that the average 100-grain weight of Chinese soybean varieties (17.42 g) was significantly higher than the average 100-grain weight of Uruguayan soybean varieties (15.31 g) (**Figure 1A**). Among the Chinese soybean varieties, CN1 exhibited the highest 100-grain weight at 24.33 g, while CN22 had the lowest at 13.66 g. Among Uruguayan soybean varieties, UY19 had the highest 100-grain weight at 18.7 g, and UY11 had the lowest at 12.33 g.

The result of the determination of the protein contents of the Sino-Uruguayan soybean germplasm showed that the quality characteristics of the Chinese soybean resources were characterized by a high protein content (range of variation 38.74% to 45.65%, mean value 42.81%) significantly higher than the Uruguayan soybean varieties. The range of variation of protein content of Uruguayan soybeans was 38.36% to 42.06%, with an average of 40.45% (**Figure 1B**). The Chinese soybean resource with the highest protein content was CN2, and the Uruguayan soybean variety with the highest protein content was UY13.

By comparing the oil content of the Sino-Uruguayan soybean germplasm, it was found that the oil content of Uruguayan soybean seeds was higher, with an average of 21.44%, which was significantly higher than that of Chinese soybean seeds (19.87%) Uruguayan soybean varieties had oil content ranging from 20.45% to 22.64%, oil content in Chinese soybean varieties ranged from 18.57% to 23.08% (**Figure 1C**). The Uruguayan soybean variety with the highest oil content was UY3, and the Chinese soybean variety with the highest oil content was CN20.

The contents of total soluble sugar, fructose, glucose, sucrose, and stachyose in soybeans from China and Uruguay were measured, with significant differences observed in the sugar contents of the germplasms of the two regions (**Figure 1D**; **Supplementary Figure 1**). In Uruguayan soybeans, the total soluble sugar content ranged from 85.97 mg/g to 108.78 mg/g, with an average of 96.99 mg/g. The variety with the highest total soluble sugar content was UY23. For Chinese soybeans, the total soluble sugar content ranged from 57.62 mg/g to 101.60 mg/g, with an average of 75.40 mg/g. The variety with the highest total soluble sugar content was CN1. In Uruguayan soybeans, the fructose, glucose, sucrose, and stachyose contents ranged from 1.92 mg/g to 5.46 mg/g, 3.56 mg/g to 5.28 mg/g, 49.47 mg/g to 61.58 mg/g, and 20.72 mg/g to 35.06 mg/g, respectively. In Chinese soybeans, the fructose, glucose, sucrose, and stachyose contents ranged from 1.75 mg/g to 3.90 mg/g, 2.61 mg/g to 4.84 mg/g, 28.27 mg/g to 56.47 mg/g, and 15.71 mg/g to 36.84 mg/g, respectively. The total soluble sugar, fructose, glucose, sucrose, and stachyose contents in Uruguayan soybeans were significantly higher than those in Chinese soybeans.

### 3.2 Genetic diversity and population structure analysis of Sino-Uruguayan soybean varieties

**Figure 2:**
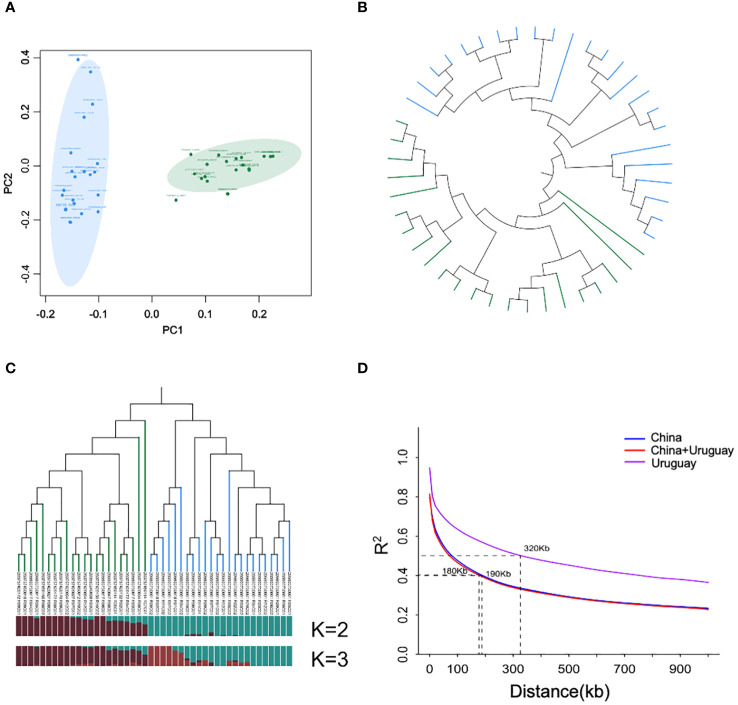
Genetic diversity between Sino-Uruguayan soybeans. **(A)** Scatter plots of principal component analysis (PCA) of Chinese (CN) and Uruguayan (UY) soybean cultivars. The green circle represents the Chinese cultivars, and the blue circle represents the Uruguayan cultivars. **(B)** Neighbor-joining trees of soybean varieties. Green and blue branches represent Chinese and Uruguayan varieties, respectively. **(C)** Genetic structure of Sino-Uruguayan soybean cultivars. The tree branches in green and blue represent Chinese cultivars and Uruguayan cultivars, respectively. Population structure at K = 2, in which dark red represents the Chinese soybean subgroup and dark green represents the Uruguayan soybean subgroup. **(D)** Comparison of linkage disequilibrium (LD) decay between Sino-Uruguayan soybean cultivars. The blue line represents Chinese cultivars, the purple line represents Uruguayan cultivars, and the red line represents Sino-Uruguayan soybean cultivars.

In the PCA plot, the tested soybean materials were distinctly divided into two main genetic groups, corresponding to their respective origins in China and Uruguay. The contribution rate of PC1 was 54.96% and the contribution rate of PC2 was 26.81%. The genetic differences among the Chinese population were minimal, whereas significant variations were observed among the Uruguayan population (**Figure 2A**). The evolutionary tree constructed based on the neighbor-joining (NJ) features further revealed a clear separation between the Sino-Uruguayan soybean varieties (**Figure 2B**). When K = 2, the Sino-Uruguayan soybean populations were divided into two major clusters. When K = 3, the populations were further subdivided into three clusters, with the Uruguayan population subdivided into two subclusters. One subcluster included 6 varieties, while the other included 18 varieties. The PCA confirmed the clear regional separation between the Sino-Uruguayan soybean varieties (**Figure 2B**). Consequently, K = 2 was selected as the optimal number of clusters (**Figure 2C**). Then, the average LD decay was calculated for all chromosomes in the two Sino-Uruguayan soybean subpopulations. The LD decay rate varied between the subpopulations, reaching a distance of 190 kb in the Chinese subpopulation compared to a distance of 320 kb in the Uruguayan subpopulation (**Figure 2D**) (**Supplementary Table 2**).

### 3.3 Selection interval and gene ontology enrichment analysis

**Figure 3:**
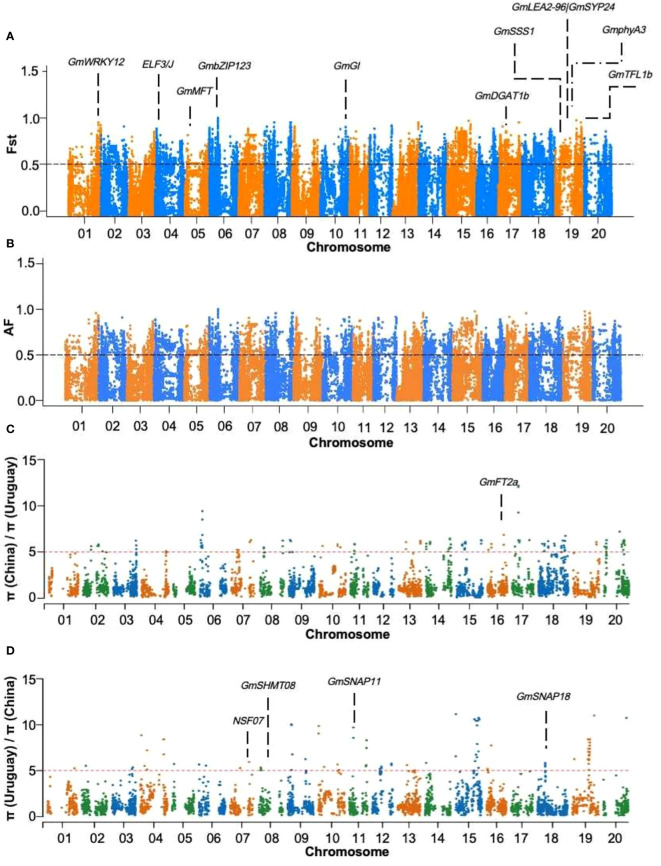
Selection signatures between Sino-Uruguayan soybeans. **(A)** Fst analysis of Sino-Uruguayan soybeans. The trait associated with the selected gene *GmWRKY12* located on chromosome 1 is drought resistance, trait associated with the selected gene *ELF3/J* located on chromosome 4 is flowering time, trait associated with the selected gene *GmMFT* located on chromosome 5 is oil content, trait associated with the selected gene *GmbZIP123* located on chromosome 6 is oil content, trait associated with the selected gene *GmGI* located on chromosome 10 is flowering time, trait associated with the selected gene *GmDGAT1b* located on chromosome 17 is oil content, trait associated with the selected gene *GmSSS1* located on chromosome 19 is seed weight, trait associated with the selected gene *GmLEA2-96|GmSYP24* located on chromosome 19 is drought resistance, trait associated with the selected gene *GmphyA3* located on chromosome 19 is flowering time, and the trait associated with the selected gene *GmTFL1b* located on chromosome 19 is growth habit. **(B)** AF analysis of Sino-Uruguayan soybeans. **(C)** Manhattan plots of the ratio of π (China)/π (Uruguay). The trait associated with the selected gene *GmFT2a* located on chromosome 16 is flowering time. **(D)** Manhattan plots of the ratio of π (Uruguay)/π (China). The trait associated with the selected genes *NSF07* located on chromosome 7, *GmSHMT08* located on chromosome 8, *GmSNAP11* located on chromosome 11, and *GmSNAP18* located on chromosome 18 is soybean cyst nematode resistance.

To identify candidate genes associated with agronomic traits, a selection elimination analysis was conducted on the 46 Sino-Uruguayan soybean germplasm using the ZDX1 soybean array. To obtain high-quality SNPs, an estimation was performed on the soybean SNP set, retaining 96,446 SNPs with a MAF greater than 5%. Candidate genes for different traits were identified based on functional annotations within the selection-affected interval. By performing selection elimination analysis on the Sino-Uruguayan soybean germplasm, the Manhattan plot based on Fst showed 2,911 selected genes that were significantly enriched at the top 5% level (**Figure 3A**). To further verify the Fst results, an allele frequency (AF) analysis was performed on the Sino-Uruguayan soybean germplasm by observing gene frequency changes within the population, and based on the Manhattan plot showed that there were 1,804 genes significantly enriched for selected genes at the top 5% level (**Figure 3B**). Nucleotide polymorphisms (π) were the main measure of genetic diversity. In the analysis of π in the Sino-Uruguayan soybean germplasms, the π value of Uruguayan soybean varieties was 502 genes selected at the top 5% level (**Figure 3C**), and the nucleotide diversity π value of Chinese soybean varieties was 384 genes selected at the top 5% level (**Figure 3D**).

For a more in-depth analysis of the functions of the selected genes, GO enrichment analysis was conducted on the genes selected (**Supplementary Table 3**). Specifically, GO enrichment was performed on genes selected through the Fst analysis, revealing 4 GO terms in the biological process (BP) category. The annotation results indicated that the variant genes were enriched in biological processes such as glycosyl compound metabolism (GO: 1901657) and nucleoside metabolism (GO: 0009116). GO enrichment of genes selected through the AF analysis yielded 48 GO terms in the BP category. The variant genes were found to be enriched in biological processes such as glycosyl compound metabolism (GO: 1901657), trehalose metabolism (GO: 0005991), glutamate metabolism (GO: 0006536), disaccharide biosynthetic processes (GO: 0046351), and oligosaccharide biosynthetic processes (GO: 0009312). The π analysis of the selected genes in Chinese soybeans, coupled with the GO enrichment analysis, revealed 3 GO terms in the BP category. The variant genes were predominantly associated with biological processes such as chloride transport (GO: 0006821) and anion transport (GO: 0006820). Similarly, GO enrichment analysis of selected genes in the Uruguayan soybeans through the π analysis identified 20 enriched GO terms in the BP category. These genes were mainly related to biological processes such as dolichol-linked oligosaccharide biosynthetic processes (GO: 0006488), protein N-linked glycosylation (GO: 0006487), cellular lipid metabolism (GO: 0044255), oligosaccharide-lipid intermediate biosynthetic processes (GO: 0006490), protein glycosylation (GO: 0006486), and glycosylation (GO: 0070085). Based on the above results, it appears that the selected genes regulate multiple complex growth and development processes in soybeans, and may be involved in various metabolic and biosynthesis processes through various cellular regulatory mechanisms, thus leading to differences in seed-related characteristics, such as the protein, oil and sugar contents, between Sino-Uruguayan soybean germplasm.

### 3.4 Selected genes

In this study, a window value of 10 kb and a step size of 5 kb were employed for AF, Fst, and π analyses on the Sino-Uruguayan soybean germplasm. Within the selected intervals, 15 known functionally relevant genes were identified (**Supplementary Table 4**). Notably, *GmWRKY12* and *GmLEA2-96*|*GmSYP24* were associated with drought, while *ELF3*/*J*, *GmGI*, *GmphyA3*, and *GmFT2a* were related to flowering. *GmTFL1b* was associated with growth habits, while *GmMFT*, *GmDGAT1b*, and *GmbZIP123* were linked to oil content. *GmSSS1* was found to be associated with seed weight, while *NSF07*, *GmSHMT08*, *GmSNAP11*, and *GmSNAP18* were related to the soybean cyst nematode (SCN).

**Figure 4:**
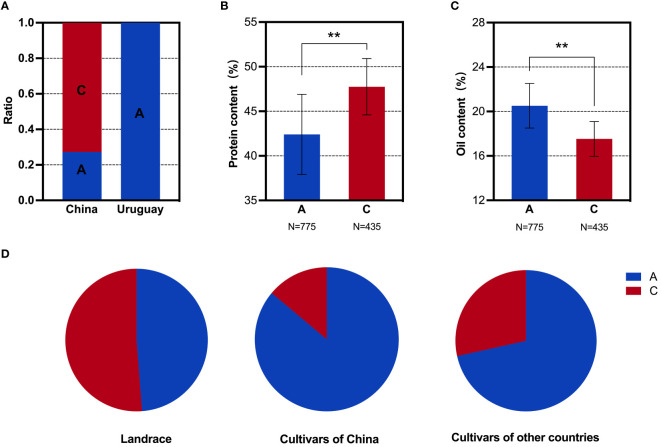
SNP analysis of the *GmMFT* locus in soybean populations. **(A)** Distribution frequencies of SNPs in the Sino-Uruguayan soybean germplasm. **(B)** Comparison of the protein contents with different SNP alleles in the 1,856 soybean accessions. **(C)** Comparison of the oil contents with different SNP alleles in the 1,856 soybean accessions. **(D)** Distribution frequencies of SNP alleles in landrace and cultivars of 1,856 soybean accessions. **, P < 0.01, according to Student's t-test.

**Figure 5:**
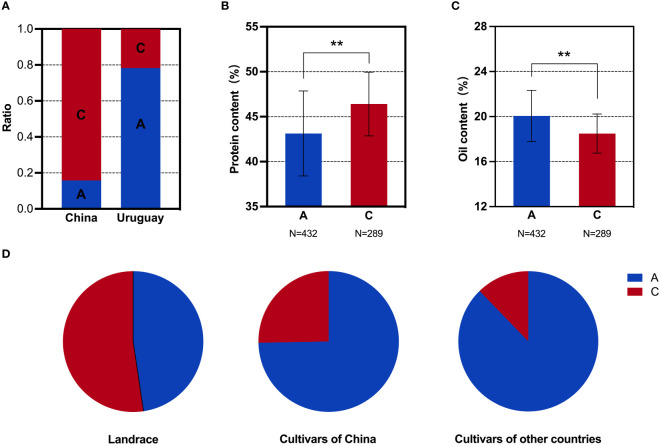
SNP analysis of the *GmDGAT1b* locus in soybean populations. **(A)** Distribution frequencies of SNPs in the Sino-Uruguayan soybean germplasm. **(B)** Comparison of the protein contents with different SNP alleles in the 1,856 soybean accessions. **(C)** Comparison of the oil contents with different SNP alleles in the 1,856 soybean accessions. **(D)** Distribution frequencies of SNP alleles in landrace and cultivars of 1,856 soybean accessions. **, P < 0.01, according to Student's t-test.

**Figure 6:**
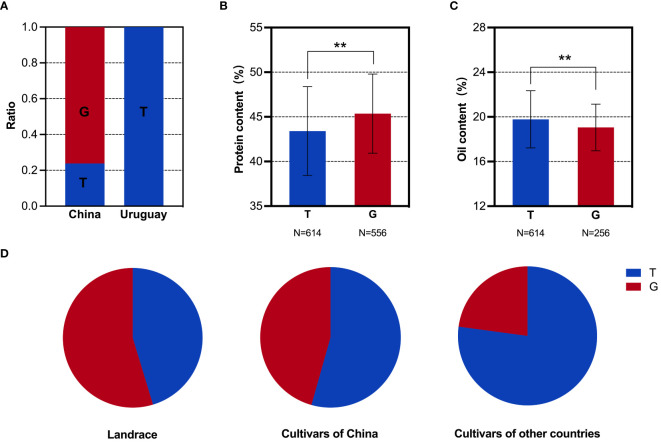
SNP analysis of the *GmbZIP123* locus in soybean populations. **(A)** Distribution frequencies of SNPs in the Sino-Uruguayan soybean germplasm. **(B)** Comparison of the protein contents with different SNP alleles in the 1,856 soybean accessions. **(C)** Comparison of the oil contents with different SNP alleles in the 1,856 soybean accessions. **(D)** Distribution frequencies of SNP alleles in landrace and cultivars of 1,856 soybean accessions. **, P < 0.01, according to Student's t-test.

To further analyze the differences in the selected genes between Sino-Uruguayan soybean germplasm, SNP allele frequency analysis was conducted on 15 selected genes (**Figures 4**–**6**; **Supplementary Figure 2**). Among these, *GmWRKY12*, *GmLEA2-96|GmSYP24*, *GmGI*, and *GmTFL1b* had both allele A and allele G in Sino-Uruguayan soybean germplasm. *ELF3/J*, *GmSSS1*, and *GmSNAP11* had allele C and allele T, while *GmSHMT08* and *GmSNAP18* contained allele C and allele G. *NSF07*, *GmphyA3*, and *GmFT2a* had allele A and allele C, allele A and allele T, and allele G and allele T, respectively. Candidate genes associated with the oil content, seed weight, and other agronomic traits were identified within the selected genomic regions. Notable genes included: *GmbZIP123* which is involved in seed oil biosynthesis. Different allelic variants of this gene were associated with variations in oil content between the Sino-Uruguayan soybean varieties. Further, *GmMFT*, which participates in the regulation of the seed oil content. Significant differentiation in haplotype frequencies between Sino-Uruguayan soybean germplasm was observed. In addition, *GmDGAT1b*, which influences the fatty acid composition by increasing the oleic acid levels. Allele frequency differences between Sino-Uruguayan soybean germplasm contributed to oil content variation. Finally, *GmSSS*1, which is associated with the soluble sugar content of seeds. Variations in this gene were correlated with differences in the soluble sugar content between the two groups.

### 3.5 Oil-related genes

Based on the above phenotypic data, it was evident that the oil content in the Uruguayan soybean germplasm was significantly higher than that in the Chinese soybean germplasm. Therefore, a detailed analysis of the SNP allele frequencies of three genes within the selected interval that contributed to the difference in oil content between Sino-Uruguayan soybean germplasm was conducted (**Supplementary Table 5**). The gene *GmMFT* (Glyma.05G244100) located on chromosome 5 showed a distribution frequency of allele A of 27.27% and allele C of 72.73% in the Chinese soybean germplasm, whereas only allele A was present in the Uruguayan soybeans (**Figure 4A**). Further analysis within a resequenced population of 1,856 soybean samples, containing 775 soybean germplasm with allele A and 435 with allele C, revealed significant differences in the protein and oil contents. In the protein content analysis, allele C was significantly higher than allele A (**Figure 4B**), while in the oil content analysis, allele A was significantly higher than allele C (**Figure 4C**). Statistical analysis of *GmMFT* with alleles A and C showed that this gene was selected for high oil and low protein varieties in both Chinese varieties and globally bred soybean varieties (**Figure 4D**). *GmDGAT1b* (Glyma.17G053300) located on chromosome 17 exhibited a distribution frequency of allele A of 15.79% and allele C of 84.21% in the Chinese soybean germplasm, while in the Uruguayan soybean germplasm, allele A had a higher frequency (78.26%) and allele C had a slightly lower frequency (21.74%) (**Figure 5A**). Similar to *GmMFT*, analysis of the protein and oil contents in a resequenced population of 1,856 soybean samples, with 432 soybean germplasm having allele A and 289 allele C, revealed consistent results, with a higher protein content for allele C and higher oil content for allele A (**Figures 5B, C**). This gene was also selected for high oil and low protein varieties in both Chinese and globally bred soybean varieties (**Figure 5D**). *GmbZIP123* (Glyma.06G010200) located on chromosome 6 exhibited a distribution frequency of allele T of 23.81% and allele G of 76.19% in the Chinese soybean germplasm, while only allele T was present in the Uruguayan soybean germplasm (**Figure 6A**). Analysis of the protein and oil contents in a resequenced population of 1,856 soybean samples, with 614 soybean germplasm having allele T and 556 with allele G, revealed a higher protein content for allele G and higher oil content for allele T (**Figures 6B, C**). This gene was selected for high protein and low oil varieties in landraces and high oil and low protein varieties in both Chinese and globally bred soybean varieties (**Figure 6D**).

**Figure 7:**
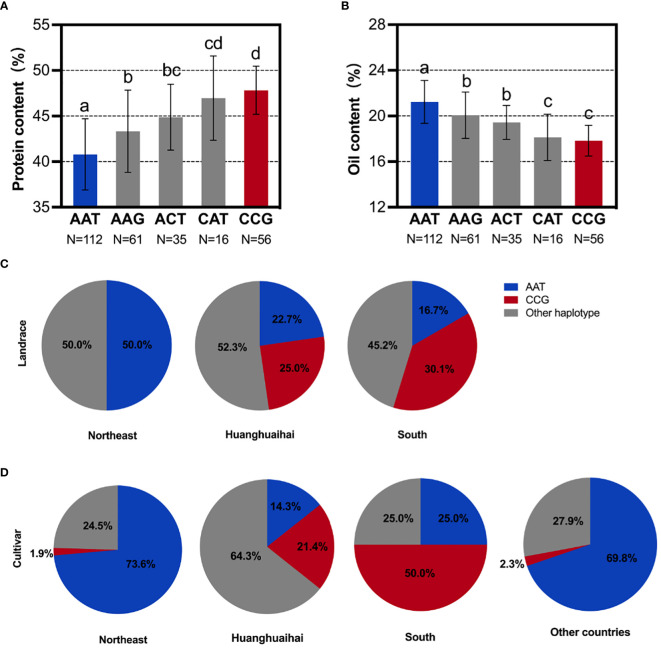
SNP analysis of the *GmMFT, GmDGAT1b, GmbZIP123* loci in soybean populations. **(A)** Comparison of the protein contents with different SNP alleles in the 1,856 soybean accessions. **(B)** Comparison of the oil contents with different SNP alleles in the 1,856 soybean accessions. **(C)** Distribution frequencies of the different SNP alleles in landrace. **(D)** Distribution frequencies of the different SNP alleles in cultivars. AAT represents the *GmMFT* gene with allele A, the *GmDGAT1b* gene with allele A, and the *GmbZIP123* gene with allele T. CCG represents the *GmMFT* gene with allele C, the *GmDGAT1b* gene with allele C, and the *GmbZIP123* gene with allele G. The same goes for the others. Error bars represent SD. Different letters indicate significant differences at P < 0.05.

To further confirm that the difference in the oil contents between the Sino-Uruguayan soybean germplasm is attributed to these three genes (*GmMFT*, *GmDGAT1b*, and *GmbZIP123*), a differential analysis of the protein and oil contents was conducted on varieties with haplotypes AAT, AAG, ACT, CAT, and CCG in a resequenced population of 1,856 soybean samples. In the protein content differential analysis, the results revealed that the CCG haplotype was significantly higher than the AAT haplotype (**Figure 7A**), while in the oil content differential analysis, the AAT haplotype was significantly higher than the CCG haplotype (**Figure 7B**). Therefore, the AAT haplotype increased the oil content, while the CCG haplotype increased the protein content. In Chinese landraces, the haplotype distribution revealed that in the Huanghuaihai and southern varieties, the CCG haplotype was more prevalent than the AAT haplotype, whereas in northeastern soybeans, the AAT haplotype was more predominant (**Figure 7C**). This suggests that in Chinese landraces, the selection trend is for a higher protein content in soybeans from the Huanghuaihai region and southern varieties, while northeastern soybeans exhibit a higher oil content. For inbred varieties, the haplotype distribution of AAT and CCG was consistent with the overall trend in Chinese landraces. Specifically, northeastern soybeans and soybeans from other countries predominantly had the AAT haplotype, while soybeans from the Huanghuaihai region and southern varieties had a higher proportion of the CCG haplotype (**Figure 7D**). This indicates that for inbred varieties, the selection trend was for higher oil contents in northeastern soybeans and soybeans from other countries, while southern Chinese soybeans had a higher protein content. Given the phenotypic data presented above, where Uruguayan soybeans were mostly high-oil and low-protein varieties, these results suggest that to achieve complementary advantages between the Sino-Uruguayan soybean germplasm, it may be beneficial to cultivate high-protein varieties similar to those found in the Huanghuaihai region of southern China and in Uruguay.

## 4 Discussion

### 4.1 Phenotypic differences between Sino-Uruguayan soybeans

Most agronomic traits are easily influenced by environmental factors. This means that phenotypic data are unable to accurately reflect the genetic diversity of entire populations (Hu et al., 2000). The oil content is controlled by both environmental and genetic factors (Pathan et al., 2013). It has been shown that soybean kernels grown in Turkey differ in oil content, with the oil content variability ranging from 18.29% to 23.06%; the highest oil content was found in the Suluova and Bafra regions (Arslanoglu et al., 2011). Additionally, research by Teng et al. (2017) revealed significant differences in soybean grain oil contents among different varieties grown in China. In this study, a significant difference in the oil content between Sino-Uruguayan soybean germplasm was observed, with Uruguayan soybeans having a markedly higher oil content than Chinese soybeans.

The protein content of soybean seeds varies as a function of genotype and may be influenced by environmental conditions, planting locations, and sowing conditions (Li et al., 2004). In China, soybeans planted in the Huanghuai region and southern multi-cropping areas have higher protein contents than soybeans planted in northeastern China (Liu J. et al., 2017; Wang et al., 1998). Rotundo et al. (2016) found that soybeans grown in the southern United States generally have a higher protein content than those grown in the north. In contrast, this study compared the protein quality of Sino-Uruguayan soybeans in the same environment and found that the protein content of Uruguayan soybean germplasm was still significantly lower than that of Chinese soybeans This suggests that differences in genotypes may lead to differences in protein content.

Qi et al. (2022) found that the main components of soluble sugars in soybean grains are correlated with latitude, with the geographical origin of soybean grains affecting the composition of soluble sugars. Matei et al. (2017) conducted a sugar content analysis of soybeans from Brazil and revealed a strong genotype and environment interaction for sucrose, glucose, raffinose, and stachyose contents in Brazilian environments. The results of this study also suggest that the significant differences in sugar contents between Sino-Uruguayan soybean germplasm may be due to genotype variations. Azam et al. (2021) analyzed phenotypic traits in soybean germplasms from China and the United States, showing that the average oil content (20.5%) and soluble sugar content (109.0 mg/g) in American soybeans were higher than those in Chinese soybeans (average oil content of 19.5% and soluble sugar content of 107.4 mg/g), while the average protein content in American soybeans (40.2%) was lower than that in Chinese soybeans (42.4%). Consistent with these findings, our study showed significant differences in protein, oil, and soluble sugar traits between Sino-Uruguayan soybean germplasm, with a higher protein content in Chinese soybeans and higher oil and total soluble sugar contents in Uruguayan soybeans. Additionally, this study identified differences in genes related to drought resistance, flowering, and SCN traits between the Sino-Uruguayan soybean populations, warranting further investigation into whether these differences result in observable phenotypic variations.

### 4.2 Genetic structure and gene differentiation as the basis of phenotypic variation

China, as the center of origin of soybeans, possesses rich soybean resources. Soybean germplasm resources disseminated from China have facilitated the breeding of new soybean varieties worldwide, contributing significantly to the development of the global soybean industry and sustainable protein production (Guo et al., 2022). Soybean cultivation in different countries leads to distinct genetic foundations. An analysis by Potapova et al. (2023) of the population structure of 103 soybean accessions from western Siberia and 72 soybean accessions from Russia revealed four taxa, with two clusters primarily composed of samples from breeding varieties in western Siberia and the third and fourth clusters containing varieties from the Far East and Europe, respectively. Nawaz et al. (2021) clustered 297 wild seed samples from Southeast Asia (89 from China, 95 from Korea, 97 from Japan, and 16 from far East Russia) into three clusters representing China, Japan, and Korea. Additionally, an analysis of 207 seed samples from different countries representing the European gene bank showed that genetic diversity was broader in United States soybean varieties and Austrian soybean varieties but narrower in Swiss and Croatian soybean varieties. A cluster analysis revealed two main clusters, consistent with the results of the structure analysis (Andrijanić et al., 2023). Moreover, Yamanaka et al. (2007) conducted a cluster analysis of 272 soybean varieties (194 from China, 59 from Japan, and 19 from Brazil), revealing that Chinese and Japanese soybean varieties were not independent but had relatively distant genetic distances, while Brazilian varieties were genetically distant from varieties from the other two countries, forming a cluster with a greater genetic distance from the gene bank of the other two countries. Furthermore, Maldonado Dos Santos et al. (2022) found that the genetic basis of Brazilian soybean varieties was narrower than that of United States soybean varieties in a study involving 235 Brazilian soybean varieties and 675 United States soybean varieties. The population structure analysis of the phenotypic traits of 46 Sino-Uruguayan soybean varieties in Sino-Uruguayan soybean germplasm in this study also revealed clear differentiation into two clusters, highlighting the distinct genetic foundations, genetic diversity, and population structures of soybean germplasm from different countries.

Population-specific differences in LD decay and the Chinese soybean population’s enhanced selection intensity may also contribute to these overall differences. Previous studies have suggested that Chinese local varieties generally exhibit greater genetic diversity and faster LD decay rates (Li et al., 2020). This study similarly showed that the LD decay rate in the Chinese soybean population was faster than that in the Uruguayan soybean population (**Figure 5**). Despite Sino-Uruguayan soybeans being grown in the same environment, the population structures of Sino-Uruguayan soybean varieties were distinct. The significant differences in traits such as the protein content, oil content, 100-grian weight, and soluble sugar content between Sino-Uruguayan soybean varieties confirm the effects of natural and artificial selection. Genetic loci associated with the traits under selection could lead to phenotypic differences between populations from the two countries. This study identified differences in allelic frequencies in genes such as *GmbZIP123*, *GmDGAT1b*, *GmMFT*, and *GmSSS1*, contributing to changes in resources between Sino-Uruguayan soybean germplasm. The genetic diversity of soybeans in China and Uruguay needs to be further expanded in future breeding work, in order to provide a basis for the innovative utilization of soybean germplasm in the future.

### 4.3 Identification of new genes

During soybean domestication, certain genes associated with specific traits were selected, leading to a significant reduction in region-specific genetic diversity. A selective sweep analysis can be employed to locate these selected regions, and, in conjunction with previously identified loci, can help infer differences between populations or aid in the discovery of new genes (Wen et al., 2015; Wang et al., 2017). The results of this study revealed substantial genetic differences between Sino-Uruguayan soybean germplasm. A selective sweep analysis, utilizing Fst, π, and AF, was conducted on the 46 Sino-Uruguayan soybean germplasm. This analysis minimizes false positives, facilitating the localization and genetic analysis of regions. Furthermore, due to the limited number of chip sites used in this study, resequencing was needed to enrich this variation. In addition, this study revealed significant differences in seed-related traits such as the oil content, protein content, 100-grian weight, and soluble sugar content, and identified genes associated with these traits within the selected interval: *GmWRKY12*, *GmLEA2-96|GmSYP24*, *ELF3/J*, *GmGI*, *GmphyA3*, *GmTFL1b*, *GmMFT*, *GmbZIP123*, *GmDGAT1b*, *GmSSS1*, *GmFT2a*, *NSF07*, *GmSHMT08*, *GmSNAP11*, and *GmSNAP18*. This provides a basis for exploring new genes.

Zhu et al. (2022) found that *GmSSS1* (Glyma. 19G196000), a gene controlling grain size in soybeans, had an increased 100-grain weight when glutamate was replaced with glutamine at residue 182 of its encoded protein. This study also showed that the *GmSSS1* (Glyma. 19G196000) gene controlled the 100-grain weight. A genome-wide association study of 1800 soybean germplasm resources by Duan et al. (2022) revealed that the natural allelic variation in *GmST05* (Seed Thickness 05) in soybean germplasm mainly controlled the seed thickness and size. To date, several types of transcription factors identified through gene-gene co-expression analysis (including zinc finger, Dof, and bZIP types) have been proven to regulate lipid biosynthesis in soybean seeds (Song et al., 2013; Li et al., 2017; Lu et al., 2021). The current study found that *GmbZIP123* is a gene related to oil content control in Sino-Uruguayan soybean germplasm. Studies have revealed significant correlations between the oil content and high-oil alleles, as well as latitude. Oil accumulation is environmentally related and achieved through critical genes involved in oil biosynthesis modification (Zhang S. et al., 2023). A recent study by Torabi et al. (2021) discovered that down-regulation of the Glyma.17G053300 (*GmDGAT1b*) gene in the target interval increased the oleic acid level, affecting the fatty acid composition of seeds. Analysis of the genes affecting the oil content of 46 Sino-Uruguayan soybean germplasm identified Glyma.17G053300 (*GmDGAT1b*) as one of the crucial genes contributing to differences in the oil content. Additionally, Zhong et al. (2023) found that the expression levels of Glyma.17G053300 were higher in the early-maturing GZ1 and the late-maturing B13 during seed maturation. This gene encodes a type I diacylglycerol acyltransferase, participating in the last step of TAG biosynthesis. Genetic function, genomic, and bioinformatic studies have speculated that the sugar-to-oil ratio might be adjusted, affecting the fatty acid composition of seeds (Roesler et al., 2016; Zhang G. et al., 2019; Torabi et al., 2021). Cai et al. (2023) demonstrated that increased expression of the *GmMFT* gene increased the grain oil content and weight. Haplotype analysis showed that *GmMFT* underwent selection leading to the expansion of cultivated soybean haplotype purity and enrichment of oil-favorable alleles in modern soybean varieties. The results of the present study also suggest that the *GmMFT* gene contributes to the differences in the oil contents of Sino-Uruguayan soybean germplasm. Zhang et al. (2020) showed that a dinucleotide CC deletion at the C-terminus of the G*mSWEET39* gene was strongly associated with a high oil and low protein content, indicating pleiotropic effects on the protein and oil content, and demonstrated that *GmSWEET39* has a dual function of improving the oil and protein content. *GmFT2a* is one of the 12 flowering locus T (FT) genes found in soybeans, functioning as the causal gene for the E9 locus, which controls flowering (Cai et al., 2018). The present study similarly found that the *GmFT2a* gene controls flowering-related traits in Sino-Uruguayan soybean germplasm. However, comparative analyses of the Sino-Uruguayan soybean germplasm indicated that a large number of unknown genes are still involved in the Sino-Uruguayan soybean population’s divergence. For example, the 100-grain weight of *GmSSS1* was only selected in the enriched region, but it could not explain the differences in weight alone. Hence, it is possible that new genes may be revealed through the configuration of hybrid combinations of more distantly related Sino-Uruguayan soybean germplasm. Overall, this research contributes to the understanding of soybean genetic diversity and the evolutionary processes shaping phenotypic traits. By utilizing germplasm resources from different regions, breeders can develop superior soybean varieties that can meet the demands of both local and global markets.

## 5 Conclusion

This study compared seed-related traits and genetic diversity between Sino-Uruguayan soybean germplasm, revealing significant differences in key phenotypic traits and identifying genomic regions under selection. The results provide valuable insights into the genetic basis of these traits and offer potential targets for soybean breeding programs. The higher oil and soluble sugar contents of Uruguayan varieties, combined with the genetic diversity analysis, suggest specific genes and genomic regions that can be targeted to improve soybean yield and quality. These findings underscore the importance of international germplasm exchange and collaboration for enhancing crop productivity and ensuring food security.

## Data availability statement

The original contributions presented in the study are included in the article/**Supplementary Material**. Further inquiries can be directed to the corresponding authors.

## Author contributions

CS: Data curation, Formal analysis, Investigation, Methodology, Writing – original draft, Writing – review & editing. ZZ: Data curation, Investigation, Methodology, Visualization, Writing – original draft. ML: Investigation, Resources, Writing – original draft. SC: Investigation, Writing – original draft. SZ: Investigation, Writing – original draft. BG: Investigation, Writing – original draft. YL: Investigation, Writing – original draft. ZL: Investigation, Writing – original draft. XA: Writing – review & editing, Funding acquisition, Project administration, Supervision. YG: Writing – review & editing, Formal analysis, Investigation, Resources. LQ: Conceptualization, Project administration, Writing – review & editing.
