# Supplementary material for: Comparison of grain traits and genetic diversity between Chinese and Uruguayan soybeans (Glycine max L.)
Source: Front Plant Sci. 2024 Jul 24;15:1435881. doi: 10.3389/fpls.2024.1435881 (PMC11303235; doi:10.3389/fpls.2024.1435881)
Supplement: Supplementary file 1 [file Table_1.docx]

**
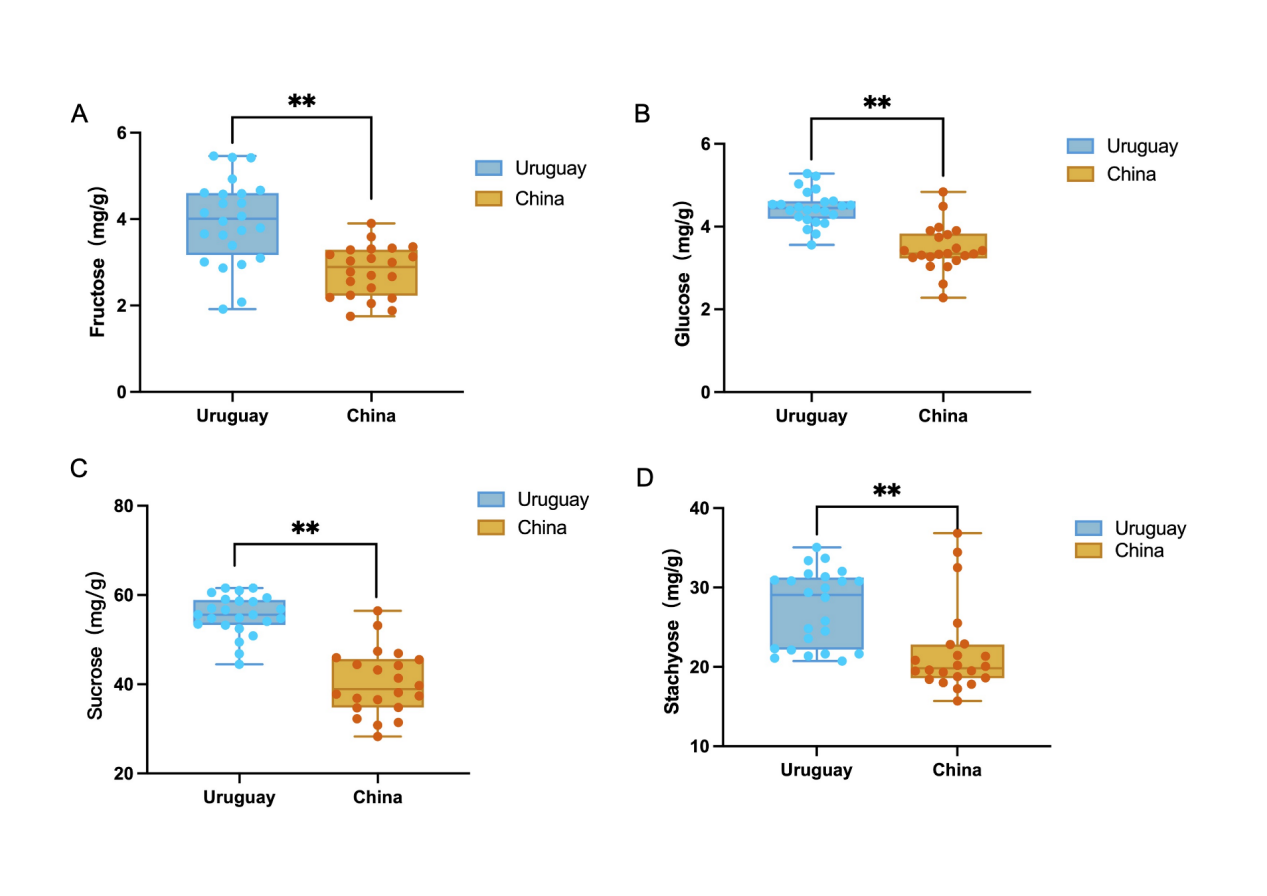
**

**Supplementary Figure 1.** Box plots of sugar content of cultivars from Uruguay and China. **(A)** Distribution of fructose. **(B)** Distribution of glucose. **(C)** Distribution of sucrose. **(D)** Distribution of stachyose. * *, P < 0.01, according to Student's t-test.
