# Supplementary material for: Comparison of grain traits and genetic diversity between Chinese and Uruguayan soybeans (Glycine max L.)
Source: Front Plant Sci. 2024 Jul 24;15:1435881. doi: 10.3389/fpls.2024.1435881 (PMC11303235; doi:10.3389/fpls.2024.1435881)
Supplement: Supplementary file 2 [file Table_2.docx]

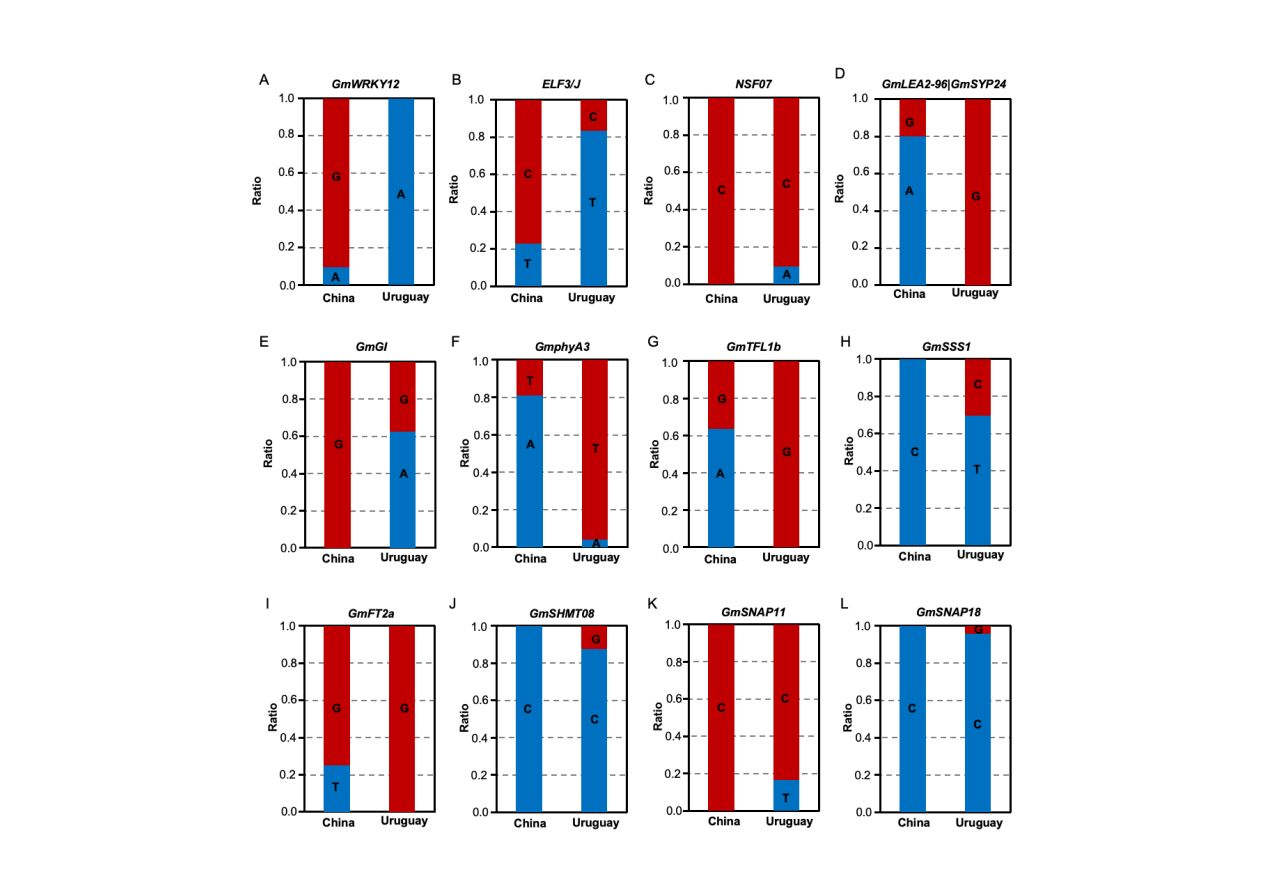


**Supplementary Figure 2.** SNPs distribution frequency of selected genes in the Sino-Uruguayan soybeans. **(A)** SNPs distribution frequency of *GmWRKY12* in soybeans in China and Uruguay. **(B)** SNPs distribution frequency of *GmLEA2-96*|*GmSYP24* in soybeans in China and Uruguay. **(C)** SNPs distribution frequency of *ELF3*/*J* in soybeans in China and Uruguay. **(D)** SNPs distribution frequency of *GmGI* in soybeans in China and Uruguay. **(E)** SNPs distribution frequency of *GmphyA3* in soybeans in China and Uruguay. **(F)** SNPs distribution frequency of *GmTFL1b* in soybeans in China and Uruguay. **(G)** SNPs distribution frequency of *GmSSS1* in soybeans in China and Uruguay. **(H)** SNPs distribution frequency of *GmFT2a* in soybeans in China and Uruguay. **(I)** SNPs distribution frequency of *NSF07* in soybeans in China and Uruguay. **(J)** SNPs distribution frequency of *GmSHMT08* in soybeans in China and Uruguay. **(K)** SNPs distribution frequency of *GmSNAP11* in soybeans in China and Uruguay. **(L)** SNPs distribution frequency of *GmSNAP18* in soybeans in China and Uruguay.
